# Non-Coding RNAs Including miRNAs and lncRNAs in Cardiovascular Biology and Disease

**DOI:** 10.3390/cells3030883

**Published:** 2014-08-22

**Authors:** Masaharu Kataoka, Da-Zhi Wang

**Affiliations:** 1 Department of Cardiology, Boston Children’s Hospital, Harvard Medical School, 320 Longwood Avenue, Boston, MA 02115, USA; 2 Department of Cardiology, Keio University School of Medicine, Shinanomachi 35, Shinjuku-ku, Tokyo 160-8582, Japan; 3 Harvard Stem Cell Institute, Harvard University, Cambridge, MA 02138, USA

**Keywords:** cardiac disease, heart development, long non-coding RNAs, microRNAs

## Abstract

It has been recognized for decades that proteins, which are encoded by our genome and produced via transcription and translation steps, are building blocks that play vital roles in almost all biological processes. Mutations identified in many protein-coding genes are linked to various human diseases. However, this “protein-centered” dogma has been challenged in recent years with the discovery that the majority of our genome is “non-coding” yet transcribed. Non-coding RNA has become the focus of “next generation” biology. Here, we review the emerging field of non-coding RNAs, including microRNAs (miRNAs) and long non-coding RNAs (lncRNAs), and their role in cardiovascular function and disease.

## 1. Introduction

When the human genome project was completed, it was surprising that only about 20,000 to 25,000 protein-coding genes exist in our species, with less than 2% of the human genome used for coding proteins. What are the functions of non-coding sequences, which make up more than 98% of our genome? The answers are now emerging with the recognition that the majority of the genome is actively transcribed to produce thousands of non-coding transcripts, including microRNAs (miRNAs) and long non-coding RNAs (lncRNAs), in many cell types and tissues. miRNAs are a class of small non-coding RNAs (~22 nucleotides) and were first discovered in *C. elegans* two decades ago. More than 2,000 miRNAs have been found in humans, and many of them are evolutionarily conserved. By imperfect base pairing with target mRNAs in a sequence dependent manner, miRNAs repress gene expression by degrading target mRNAs and/or inhibiting their translation. Roles for miRNAs have been demonstrated in the regulation of a broad range of biological activities and diseases [1]. More recently, thousands of lncRNAs, which are transcribed non-coding RNAs greater than 200 nucleotides, were discovered and implicated in a variety of biological processes [2,3]. Clearly, investigating and understanding of how miRNAs and lncRNAs regulate gene expression during cardiovascular development and function will greatly facilitate therapeutic treatment of cardiovascular disease. Here, we briefly review the function of miRNAs and lncRNAs in the cardiovascular system and related human disorders.

## 2. miRNAs in Cardiac Development

Global disruption of the expression and maturation of all miRNAs in the heart is the first step to understand the function of miRNAs in cardiac development and physiology. Dicer, an RNase III endoribonuclease, is a critical enzyme for the maturation of most miRNAs. Conventional deletion of Dicer causes early embryonic lethality in mice, demonstrating the critical role of miRNAs in animal development [4]. Disrupting miRNA expression in early embryonic hearts using Nkx2.5-Cre mediated Dicer mutation leads to improperly compacted ventricular myocardium in mutant embryos [5], further indicating that miRNAs are indispensible for cardiogenesis. Similarly, α-MHC-Cre-mediated conditional deletion of Dicer causes postnatal lethality due to dilated cardiomyopathy and heart failure [6]. These studies suggest that many miRNAs play crucial roles in cardiac development and physiological function. miR-1 is tissue-specifically expressed in the heart and skeletal muscle, and genetic deletion of both miR-1-1 and miR-1-2 indicated that miR-1 is required for cardiomorphogenesis and the expression of many cardiac contractile proteins [7,8].

## 3. Cardiac Regeneration Regulated by miRNAs

Mammalian adult cardiomyocytes are terminally differentiated cells that exit the cell cycle. Lack of regenerative ability of adult hearts is one of the major causes of cardiomyopathy. A recent report identified about 40 miRNAs that strongly enhanced cell proliferation in neonatal mouse and rat cardiomyocytes. Two of these miRNAs, miR-590 and miR-199a, were further demonstrated to induce cardiomyocyte proliferation both *in vitro* and *in vivo* [9]. It remains to be determined which molecular target(s) mediate the function of these miRNAs in cardiomyocyte proliferation. Using both gain- and loss-of function approaches in transgenic and knockout mouse models, we demonstrated that the miR-17-92 cluster is required for and sufficient to induce cardiomyocyte proliferation. More specifically, we identified miR-19a/b as the major contributors among the miR-17-92 cluster to the regulation of the cardiomyocyte proliferation [10]. Mechanistically, we identified Pten as one of the miR-19a/19b targets which participate in the regulation of cardiomyocyte proliferation. These studies demonstrate that miRNAs are key regulators of cardiomyocyte proliferation and heart regeneration, suggesting their significant therapeutic potential to treat cardiac-degeneration associated heart disease.

In rodent hearts, neonatal cardiomyocytes can still proliferate, leading to the regeneration potential in newborn hearts. However, proliferation potential is gradually lost by postnatal day 7 (P7) and cardiomyocytes exit the cell cycle by then. As a result, hearts beyond P7 lost their potential to regenerate in response to stress or injury. Through profiling and comparing miRNA expression between P1 and P10 rat cardiomyocytes, members of the miR-15 family, including miR-195, miR-15a, miR-15b, miR-16, and miR-497, were identified as important regulators of postnatal cardiomyocyte mitotic arrest [11]. Further studies demonstrate that cardiomyocyte proliferation can be inhibited by this family of miRNAs through the repression of multiple cell cycle regulators. Interestingly, the expression of miR-15 family was also shown to be up-regulated in cardiac ischemia and heart failure [12]. Recently, it has been reported that neonatal mammalian hearts can regenerate after myocardial infarction through the proliferation of preexisting cardiomyocytes, and that the miR-15 family of miRNAs participates in this process in neonatal hearts [13]. Furthermore, it was reported that miR-15 induces apoptosis by targeting anti-apoptotic factor Bcl2 [14]. Together, these studies suggest that the miR-15 family may play distinct roles in cardiomyocyte proliferation, apoptosis under different developmental and/or pathological conditions, implying their potential to treat cardiac regeneration related disease.

Recently, an exciting breakthrough was achieved in which Ieda *et al.* developed a new strategy to directly reprogram fibroblasts into cardiomyocytes through the combination of three cardiac-specific transcriptional factors, Gata4, Mef2c, and Tbx5 (GMT) *in vitro* [15]. The above observation was further supported by two independent studies, in which Qian *et al.* and Song *et al.* demonstrated that cardiomyocyte direct reprogramming was also achievable *in vivo* [16,17]. These investigators reported that they were able to use three (GMT) or four cardiac transcription factors, Gata4, Hand2, Mef2c, and Tbx5 (GHMT) respectively, to reprogram cardiac fibroblasts into beating cardiomyocyte-like cells. More importantly, reprogramming cardiac fibroblasts into cardiomyocytes *in vivo* was shown to improve cardiac function and reduce cardiac fibrosis in a mouse model of myocardial infarction. It is not known whether miRNAs are involved in the process of reprogramming in these studies. However, it was reported in a separated study that a combination of miR-1, miR-133, miR-208, and miR-499 was able to directly induce the cellular reprogramming of fibroblasts into cardiomyocyte-like cells *in vitro* [18]. In this study, the investigators showed that miR-1 alone is sufficient to induce the fibroblast to cardiomyocyte reprogramming. However, this reprogramming efficiency was dramatically enhanced when miRNAs 133, 208, and 499 were added. Interestingly, the process of reprogramming was further enhanced by about 10-fold after JAK inhibitor I treatment. Moreover, administration of miRNAs into ischemic mouse myocardium resulted in direct conversion of cardiac fibroblasts to cardiomyocytes *in situ*. Recently, Nam *et al.*, used a combination of transcription factors and miRNAs to induce direct reprogramming of fibroblasts into cardiomyocyte-like cells [19]. They treated human fibroblasts with four transcriptional factors, GATA-4, Hand2, Tbx5 and Myocardin [20], together with two miRNAs, miR-1 and miR-133. A portion of the treated human fibroblasts was reprogrammed into cells with sarcomere-like structures, showing spontaneous contractility after 4 to 11 weeks in culture, suggesting the success of partial reprogramming.Besidesphenotypic changes, the investigators found that the transcriptome of reprogrammed cells had also shifted toward that of cardiomyocytes. Taken together, these studies indicated that miRNAs could function in concert with cardiac transcriptional factors and other signaling pathways to synergistically enhance cardiomyocyte reprogramming.

## 4. miRNAs in Cardiac Hypertrophy, Remodeling and Heart Failure

Cardiac remodeling, which is defined as an alteration in the structure (dimensions, mass, shape) of the heart, is one of the major responses of the heart to biomechanical stress and pathological stimuli. Numerous studies have demonstrated the functional involvement of many miRNAs during cardiac remodeling [21]. Cardiac hypertrophy is anatomically characterized as an increase in the thickness of the cardiac ventricular wall, owing to the enlargement of myocyte size and/or increased fibrosis. Sustained cardiac hypertrophy often leads to end stage heart failure. To investigate the involvement of miRNAs in this process, genome-wide profiling of miRNA expression has been performed and dysregulated miRNAs were identified during cardiac remodeling [22,23]. For instance, miR-21 was shown to promote cardiac fibroblast survival and the development of cardiac fibrosis by enhancing ERK-MAP kinase activity through the down-regulation of Sprouty homologue 1, a direct target of miR-21 and an endogenous inhibitor of ERK-MAP kinase [24]. Inhibition of miR-21 via an antagomir was shown to repress cardiac hypertrophy and fibrosis *in vivo* in response to stress. However, these results could not be verified through genetic deletion of miR-21 in mice [25], indicating that miR-21 may not be essential for the pathological remodeling of the heart. Most recently, it was reported that cardiac fibroblasts secrete star miRNA-enriched exosomes and identify fibroblast-derived miR-21* as a paracrine signaling mediator of cardiomyocyte hypertrophy that has potential as a therapeutic target [26]. Another study showed that isoproterenol-induced cardiac hypertrophy could be repressed when miR-23a was knocked down. miR-23a represents another miRNA up-regulated during hypertrophy and the repressive effect of miR-23a in cardiac hypertrophy was suggested, at least in part, due to the repression of MuRF1, an anti-hypertrophic factor [27].

Recently, we and others demonstrated that miR-22, a miRNA enriched in cardiomyocytes but only mildly up-regulated during cardiac hypertrophy, significantly promotes cardiac hypertrophy *in vitro* and *in vivo* [28,29]. Cardiac-specific knockout of miR-22 in mice repressed stress-induced cardiac hypertrophy, accompanied by accelerated dilation. Conversely, cardiac-specific overexpression of miR-22 induced spontaneous hypertrophic growth in the heart. Additional studies showed that miR-22 represses a broad spectrum of target genes, including Sirt1, HDAC4, PPARα, and Purb, a negative regulator of Serum Response Factor (SRF) during the regulation of cardiac hypertrophy [28,29].

In another study, we demonstrated that miR-155 is expressed in cardiomyocytes and that its expression is reduced in pressure overload-induced hypertrophic hearts [30]. In mouse models of cardiac hypertrophy, genetic deletion of miR-155 suppressed cardiac hypertrophy and cardiac remodeling in response to pathological stressors, including both transverse aortic constriction and an activated calcineurin transgene. Most importantly, we found that loss of miR-155 prevented the progress of heart failure and substantially extended the survival of calcineurin transgenic mice. These studies uncovered miR-155 as an inducer of pathological cardiomyocyte hypertrophy and suggested that inhibition of endogenous miR-155 might have clinical potential to suppress cardiac hypertrophy and heart failure. Given that miR-155 is expressed in both cardiomyocytes and non-cardiomyocytes of the heart, it is important to define the role of this miRNA in myocyte *vs.* non-myocyte portions of the heart in future studies. Furthermore, a recent study demonstrated that paracrine regulation of cardiac miRNAs by transplanted bone marrow progenitor cells contributes to the anti-fibrotic effect. Mechanistically, it was found that bone marrow progenitor cells release HGF, which inhibits miR-155-mediated profibrosis signaling, thereby preventing cardiac fibrosis under diabetic conditions [31].

Cardiac fibrosis, which is defined as abnormal deposition of collagen by cardiac fibroblasts, is often observed to replace the “drop-out” of cardiomyocytes during cardiac remodeling. Many genes and molecular pathways have been reported to participate in the regulation of this process. It is not surprising that miRNAs were reported to regulate cardiac fibrosis in recent years. Connective tissue growth factor (CTGF) is a key molecule in the process of fibrosis and therefore seemingly serves as an attractive therapeutic target [32]. However, it was unknown how CTGF transcripts were regulated post-transcriptionally. Duisters *et al.* showed that miR-133 and miR-30 were involved in myocardial matrix remodeling through regulating CTGF [33]. Both miR-133 and miR-30 were found consistently down-regulated in several models of heart failure and pathological hypertrophy. Knockdown of these miRNAs resulted in a strong increase of CTGF levels. Conversely, overexpression of miR-133 and miR-30c repressed the production of collagens, which was accompanied with a decrease in CTGF expression levels. In another study, the miR-29 family, which is predominantly expressed in cardiac fibroblasts [34], was found to be significantly down-regulated in the fibrotic border zone of infracted hearts. Intriguingly, many of the miR-29 downstream target genes, such as FBN1, COL1A1, COL1A2, ELN and COL3A1, are up-regulated after myocardial infarction, suggesting that miR-29 controls the physiological levels of many matrix proteins in such a manner that down-regulation of miR-29 is associated with an excessive accumulation of matrix protein and cardiac fibrosis. Though the function of miR-29 in cardiac fibrosis was established using gain- and loss-of function studies *in vitro* and *in vivo*, genetic evidence is still lacking to support the conclusion. Taken together, emerging evidences have demonstrated that miRNAs are not only important for cardiovascular development, but also essential factors for cardiac hypertrophy and remodeling.

miRNAs also play important roles in endothelial function, vascular integrity, and angiogenesis. Endothelial cell-restricted miR-126 mediated developmental angiogenesis via enhancement of the pro-angiogenic actions of VEGF and FGF and promoted blood vessel formation by repressing the expression of Spred-1, an intracellular inhibitor of angiogenic signaling [35]. miR-24 is enriched in cardiac endothelial cells and upregulated after cardiac ischemia, and acts as a critical regulator of endothelial cell apoptosis and angiogenesis [36]. Furthermore, it has been reported that miR-24 suppression prevents the transition from compensated hypertrophy to decompensated hypertrophy by stabilizing junctophilin-2 expression and protecting the ultrastructure of T-tubule-sarcoplasmic reticulum junctions [37]. Similarly, miR-210 can improve angiogenesis, inhibit apoptosis, and improve cardiac function in a murine model of myocardial infarction, though the molecular mechanism is not fully understood [38].

## 5. Cardiac Ischemia Regulated by miRNAs

Ischemia is an independent risk factor of cardiovascular events, which leads to myocardial infarction and ischemia-reperfusion injury. At cellular level, cardiomyocytes often undergo apoptosis following myocardial infarction and ischemia-reperfusion injury. Several miRNAs participate in the regulation of these pathologic processes. miR-92a, a member of the miR-17-92 cluster involved in cardiomyocyte proliferation, also participates in the control of cardiomyocyte survival by targeting integrin subunit α5 and eNOS. Inhibition of miR-92a by antagomir improved cardiac function and reduced cardiomyocyte apoptosis after MI in mice [39]. miR-21 serves as an anti-apoptotic factor in myocardial infarction animal models by targeting PDCD4 and repressing its expression. Interestingly, miR-21 seems to target cardiac fibroblasts, not cardiomyocytes, in the early phase of acute myocardial infarction, highlighting the significant contribution of cardiomyocyte-fibroblast interaction to cardiac function [40]. Conversely, miR-320 is down-regulated after ischemia-reperfusion injury. Gain- and loss-of-function studies demonstrated that miR-320 promotes cardiomyocyte apoptosis via maintaining HSP20 levels [41]. It is speculated that additional miRNAs will be found participating in the regulation of cardiac ischemia and heart disease.

## 6. miRNAs “Conducting” Arrhythmia

The cardiac conduction system can be damaged following cardiac injury, such as cardiac ischemia or acute MI, and cellular necrosis can lead the dysfunction of the whole cardiac conduction system, including the sinoatrial node, atrioventricular node, and His-Purkinje system. Electrical signals cannot be conducted smoothly through this damaged conduction system, resulting in a series of arrhythmia. miRNAs have been shown to participate in this process and the proper expression of miRNAs is critical for sustaining the normal function of cardiac conduction system. For instance, it has been reported that miR-1 and miR-133, two most commonly expressed miRNAs in striated muscle, target several ion channel and gap-junction associated genes, such as HCN2, HCN4, KCNJ2, ERG and GJA1 (Cx43) [42]. Overexpression of miR-1 in infarcted myocardium can promote arrhythmogenesis, whereas arrhythmia could be alleviated through deleting endogenous miR-1. miR-208a, a cardiac-specific miRNA encoded by the intron of the myosin heavy chain gene *Myh6*, has also been demonstrated to play an important role in arrhythmogenesis [43], especially in the process of atrial depolarization, by regulating the expression of Connexin-40 (GJA5). Therefore, studies have established the role of miRNAs in the development and maintenance of the cardiac conduction system.

## 7. Diagnostic and Therapeutic Uses of miRNAs

miRNAs could be utilized as biomarkers for the diagnosis of cardiovascular disease, given that the expression of many miRNAs is altered in a variety of cardiac biologic processes and disease conditions. Importantly, “circulating miRNAs” appear to be stable in mammalian serum and plasma, which are easy to acquire, raising the possibility that they could serve as biomarkers for heart disease diagnosis and prediction [44,45]. Recently, a clinical study has already made progress toward this possibility by showing that circulating miR-192 levels are correlated with the development of ischemic heart failure after acute myocardial infarction in human patients [46]. Furthermore, it has been reported that several circulating miRNAs, such as miR-133, miR-1291, miR-663b, miR-328, and miR-134, exhibit clinical impact on human myocardial infarction [47,48]. Likewise, miR-328 can be a potential mediator of atrial remodeling and atrial fibrillation [49].

Though the therapeutic potential of miRNAs in cardiovascular disease remains debatable, much remarkable progress in miRNA-based translational medicine has been made. Cardiologists are now attempting to use miRNAs and/or their inhibitors to treat heart diseases. In addition, many efficient techniques to manipulate miRNA levels *in vivo* have been developed. Among these techniques, antagomirs, which knock down targeted miRNAs by sequestering them from the functional complex, were shown to be stable in blood [50]. Conversely, miRNA mimics, synthesized chemically modified double-stranded oligonucleotides, can create gain-of-function effects for specific miRNAs *in vitro* and *in vivo* [51,52]. With the identification of miRNAs and their targets in normal and diseased hearts and the understanding of their function (Table 1), we are confident that diagnostic tools and therapeutics based on miRNAs will play even more important roles in the field of cardiology.

**Table 1:** miRNAs and their targets implicated in the function of the heart.

microRNA	miRNA Targets	Function in the Heart	Reference(s)
miR-1	RhoA, Cdc42, Nelf-A/WHSC2, Kcnj2, Gja1, Ppp2r5a, Vegfa	cardiac hypertrophy, arrythmia, reprogramming of fibroblasts into cardiomyocyte-like cells	[18,42,53,54]
miR-15	Chek1	postnatal cardiomyocyte mitotic arrest	[11]
miR-16	Chek1	postnatal cardiomyocyte mitotic arrest	[11]
miR-19	Pten	cardiomyocyte proliferation	[10]
miR-21	Ppara, Mpv17l, Sorbs2, Pdlim5, PDCD4	ischemia/reperfusion, cardiac hypertrophy	[24,25,40]
miR-22	Sirt1, HDAC4, PPARα, Purb	cardiac hypertrophy	[28,29]
miR-23	MuRF1	cardiac hypertrophy	[27]
miR-25	Serca2, Ip3r1	arrythmia, heart failure	[55]
miR-26	KCNJ2	arrythmia	[56]
miR-29	Col1a1, Col1a2, Col3a1, ELN	arrythmia, cardiac fibrosis	[34]
miR-30	CTGF	cardiac fibrosis	[33]
miR-34	PPP1R10, vinculin, Sema4b, Pofut1, Bcl6	myocardial infarction, cardiac hypertrophy	[57,58]
miR-132	FoxO3	cardiac hypertrophy	[59]
miR-133	CTGF	cardiac fibrosis, cardiac hypertrophy, cardiac remodeling, reprogramming of fibroblasts into cardiomyocyte-like cells	[18,33,47,54]
miR-155	Jarid2, Socs1	cardiac hypertrophy, cardiac fibrosis	[30,31]
miR-195	Chek1	postnatal cardiomyocyte mitotic arrest	[11]
miR-199	Dyrk1a	cardiac hypertrophy, cardiomyocyte proliferation	[9]
miR-208	Med13	cardiac hypertrophy, reprogramming of fibroblasts into cardiomyocyte-like cells	[18]
miR-212	FoxO3	cardiac hypertrophy	[59]
miR-214	Slc8a1, Bcl2l11, Ppif	cardiac remodeling	[60]
miR-320	Hsp6b	myocardial ischemia	[41]
miR-328	Cacna1c, Cacnb1	atrial fibrillation	[61]
miR-486	Pten, Foxo1a	cardiac hypertrophy	[62]
miR-497	Chek1	postnatal cardiomyocyte mitotic arrest	[11]
miR-499	Calcineurin, Drp1	cardiac hypertrophy, reprogramming of fibroblasts into cardiomyocyte-like cells	[18,63]
miR-590	unknown	cardiomyocyte proliferation	[9]

## 8. Long Non-Coding RNAs (LncRNAs) in Cardiac Development

Long ncRNAs (lncRNAs) are a novel class of ncRNAs that are larger than 200 nucleotides but do not encode proteins. Thousands of lncRNAs have been identified in different species. Emerging evidence has suggested that lncRNAs have crucial roles in controlling gene expression and other cellular processes during both developmental and differentiation processes. lncRNAs regulate gene expression at the levels of epigenetic control, transcription, RNA processing, and translation. Many lncRNAs have recently been discovered and their function in a variety of biological processes is emerging. However, relatively little is known about the involvement of lncRNAs in the cardiovascular system. A novel lncRNA, *Braveheart*, has been identified as a critical regulator of cardiovascular commitment from embryonic stem cells [64,65]. *Braveheart* activates a cardiovascular gene network and functions upstream of mesoderm posterior basic helix-loop-helix transcription factor 1 (MESP1), a master regulator of a common multi-potent cardiovascular progenitor. *Braveheart* mediates the epigenetic regulation of cardiac commitment by interacting with SUZ12, a component of the polycomb repressive complex 2 (PRC2), which appears to be a common mechanism for lncRNAs’ function. *Braveheart* therefore represents the first lncRNA that defines cardiac cell fate and lineage specificity, linking lncRNAs to cardiac development and disease. However, it remains to be seen if *Braveheart* is required for normal heart development *in vivo*. *Braveheart* appears to exist as a mouse specific lncRNA, as direct sequence alignment did not identify mouse *Braveheart* homologues in other species. The expression of *Braveheart* was nicely documented in mouse ESCs and heart samples using an RNA-Seq approach. However, the potentially orthologous human and rat genomic regions were not actively transcribed. The lack of an apparent human *Braveheart* homologue raises the question of how well the lessons learned from mouse *Braveheart* will translate to human cardiovascular disease. Perhaps an undiscovered functional *Braveheart* homologue, transcribed from a different genomic locus, exists in the human genome. Nevertheless, the discovery of *Braveheart* will likely impact the cardiovascular research field.

*Fendrr*, another novel lncRNA expressed in the heart, is one of very few lncRNAs whose *in vivo* functions have been explored using mouse genetics. Two independent studies demonstrated that loss of *Fendrr* is lethal in mice. Mutant mice display a spectrum of defects, including cardiac morphogenesis, consistent with the findings that *Fendrr* is expressed in the mouse lateral plate mesoderm and developing hearts [66,67]. However, there is a difference in the phenotype severity associated with the two mutant mouse lines; while one mutant line dies embryonically around E13.75, the other one dies postnatally. One possible explanation for this discrepancy is the different targeting strategies used to remove the *Fendrr* gene. Mechanistically, *Fendrr* was shown, similarly to *Braveheart*, to interact with the PRC2 complex to modulate the epigenetic regulation of gene expression. In addition, *Fendrr* may be involved in the control of the activating H3K4me3 mark on a subset of promoters, thereby modifying the expression level of those genes. However, the mechanisms of *Fendrr*-dependent molecular events remain to be fully understood. It is expected that many more lncRNAs will be found to play important roles in cardiovascular development and function.

## 9. lncRNAs in Cardiac Disease

Given the emerging role of lncRNAs in a large spectrum of biological systems examined, it is not surprising that several recent studies have identified many lncRNAs associated with diseased hearts, both in human patients with cardiovascular disease and mouse models for human disease. In these studies, the investigators took genome-wide, next generation RNA sequencing approaches and documented many lncRNAs expressed in the heart. Most importantly, they found that expression of cardiac-expressed or circulating lncRNAs was altered in patients with cardiomyopathy or heart failure [68,69,70,71]. Future investigations will certainly link the functional characteristics of lncRNAs to maladaptive remodeling, cardiac function, cardiac regeneration and cardiovascular disease.

Recent studies also linked several lncRNAs to heart disease. *ANRIL*, an lncRNA, was identified as a risk factor for coronary disease [72]. Though it is still not fully understood how ANRIL functions, evidence suggests that this lncRNA may participate in the regulation of histone methylation [73]. Another lncRNA, MIAT (myocardial infarction-associated transcript) (or *Gomafu/RNCR2*) was identified as a risk factor associated with patients with myocardial infarction [74]. However, how MIAT controls the status of myocardial infarction remains largely unknown. Intriguingly, the genetic loci that encode MYH6 and MYH7, the main myosin heavy chain genes in cardiac muscle, appear to produce a non-coding anti-sense transcript (*Myh7-as*). *Myh7-as* transcription may regulate the ratio of *Myh6* and *Myh7*, altering the function of muscle contraction [75].

While *Braveheart* was shown to be an important lncRNA for cardiac cell fate, additional cardiac-expressed lncRNAs, in particular those selectively expressed in cardiomyocytes, remain to be identified and studied. Intriguingly, recent studies demonstrate that cardiac transcription factors and miRNAs reprogram cardiac fibroblasts into cardiomyocytes *in vitro* and *in vivo* [15,16,17,18], raising the tantalizing possibility that reprogramming strategies may be used to enhance the limited native regenerative capacity of adult mammalian hearts [76,77]. Most recently, one novel lncRNA, lincRNA-RoR, was reported to modulate the reprogramming of induced pluripotent stem cells, at least in part, by regulating the expression levels and activities of key reprogramming factors, Oct4, Sox2, and Nanog [78]. These studies suggest that lncRNAs could form a feedback loop with core TFs and miRNAs to regulate ESC maintenance and differentiation. It will be interesting to determine whether lncRNAs might also participate in cardiac regeneration or be used to stimulate cellular reprogramming to directly reprogram non-myocyte cells such as cardiac fibroblasts into cardiomyocytes.

## 10. lncRNAs as Competing Endogenous RNA

Recently, it has been reported that competing endogenous RNAs (ceRNAs) regulate the distribution of miRNA molecules on their targets and thereby impose an additional level of post-transcriptional regulation. In particular, a muscle-specific lncRNA, linc-MD1, sponges miR-133 to regulate the expression of MAML1 and MEF2C, transcription factors that activate muscle-specific gene expression. It was found that HuR, which is under the repressive control of miR-133, is derepressed due to the sponging activity of linc-MD1 on miR-133. This study therefore uncovered a feedforward positive loop involving muscle transcription factors, RNA binding proteins, miRNAs, and an lncRNA, that controls early phases of myogenesis [79]. Interestingly, the levels of linc-MD1 are strongly reduced in muscle cells of patients with Duchenne Muscular Dystrophy [80]. In another study, it was reported that cardiac apoptosis-related lncRNA (CARL) could act as an endogenous miR-539 sponge to regulate PHB2 expression, mitochondrial fission and apoptosis. Modulation of their levels may provide a new approach for tackling apoptosis and myocardial infarction [81]. Clearly, understanding this novel RNA crosstalk will lead to significant insight into gene regulatory networks and have implications in human development and disease.

Importantly, lncRNAs have unique functional and regulatory characteristics. One major finding of numerous recent studies of lncRNAs, specifically within the heart, was that lncRNAs are highly tissue-specific. Genome-wide profiling of the cardiac transcriptome after myocardial infarction revealed hundreds of novel heart-specific lncRNAs with unique regulatory and functional characteristics relevant to maladaptive remodelling, cardiac function, and possibly cardiac regeneration [68]. This finding implies that heart-specific lncRNAs have ample possibilities as targeting molecules and biomarkers relevant to cardiac development and disease.

## 11. Future Prospects

We have just started the era of “non-coding”. We are looking forward to see more and more reports on the roles of non-coding RNAs (miRNAs and lncRNAs) in the regulation of a variety of essential biological processes, including cardiovascular biology and disease. It is an exciting time to investigate the function of non-coding RNAs, and advanced technology development will certainly propel the research field forward. Many efficient techniques to manipulate miRNA levels *in vitro* and *in vivo*, such as antagomirs and miRNA mimics, have been developed for loss- and gain-of-function studies. Additionally, the rAAV9 vector has been demonstrated to have high affinity for myocardium [82], providing a powerful tool for delivering miRNA- and lncRNA-related therapeutic molecules specifically to the heart through intravenous injections. With efficient strategies for gain- and loss-of-function investigations, more fruitful work about the molecular mechanism and therapeutic application of non-coding RNAs in cardiovascular disease will emerge. We are confident that non-coding RNAs will take the central stage of cardiovascular medicine in the foreseeable future. Non-coding RNAs represent potential therapeutic targets for cardiac disease as well as attractive candidate biomarkers to be used in the clinic.
